# Increased Phospho-Keratin 8 Isoforms in Colorectal Tumors Associated with EGFR Pathway Activation and Reduced Apoptosis

**DOI:** 10.5402/2012/706545

**Published:** 2012-01-31

**Authors:** Georgia Arentz, Tim Chataway, Mark R. Condina, Timothy J. Price, Peter Hoffmann, Jennifer E. Hardingham

**Affiliations:** ^1^Department of Haematology-Oncology, The Queen Elizabeth Hospital, Woodville, SA 5011, Australia; ^2^Physiology Department, School of Medical Sciences, University of Adelaide, Adelaide, SA 5005, Australia; ^3^Flinders Proteomics Laboratory, Department of Human Physiology, Flinders University, Bedford Park, SA 5042, Australia; ^4^Adelaide Proteomics Centre, School of Molecular and Biomedical Science, University of Adelaide, Adelaide, SA 5005, Australia

## Abstract

Hyperphosphorylated keratin (K) 8 acts as a phosphate “sponge” for stress-activated protein kinases thereby inhibiting pro-apoptotic molecules and thus apoptosis. MAP kinase/ERK1 has increased activity in colorectal cancer (CRC) and is known to phosphorylate K8. The aims were to identify the K8 isoforms abundantly present in colon tumors, using 2D difference gel electrophoresis (DIGE), to identify the modifications using mass spectrometry, and to validate the differential abundance of these isoforms in tumors relative to matched normal mucosae. 2D DIGE showed 3 isoforms of K8 significantly increased in tumor ≥2-fold in 6/8 pairs. Metal oxide affinity chromatography mass spectrometry and bioinformatics were used to identify phosphorylated serine residues. Levels of PS24, PS432, and PS74 by western blotting were found to be significantly increased in tumor versus matched normal. Blocking of EGFR signaling in Caco2 cells showed a significant decrease (*P* < 0.0001) in K8 PS74 and PS432 levels by 59% and 66%, respectively, resulting in increased apoptosis.

## 1. Introduction

K8 and K18 are the major intermediate filament (IF) components of simple epithelia of the gastrointestinal tract, liver, pancreas, and mammary glands [1]. Cellular K8 interacts with K18 to form insoluble 10 nm filaments that extend from the nucleus to the internal leaflet of the plasma membrane, where they interact with desmosomes and hemidesmosomes to bridge transmembrane domain proteins via plakins [2]. Increased expression of K8/18 has been associated with metastasis and invasion in cancer [3–6]. The phosphorylation of IF proteins is of primary importance in their function, regulating assembly, disassembly, and organisation *in vitro* and *in vivo* [7, 8]. Toivola et al. (1997) showed that phosphorylation of IF is essential for the correct function of keratins, and when serine/threonine phosphatase activity is downregulated, cell junctions and the organisation of IF and microfilament assembly are disrupted. Increased phosphorylation of K8/18 results in increased solubilisation and the inhibition of subunit polymerisation [9]. The K8 residue serine 73 is known to be phosphorylated by c-Jun N-terminal kinase (JNK) and p38 kinases during cellular stress [10–12] and serine residue 431 is phosphorylated by ERK1 in response to EGFR stimulation [10, 12, 13]. (Phospho-serine (PS) 73 is subsequently referred to in this paper as PS74, similarly PS431 as PS432 and PS23 as PS24, in accordance with the nomenclature adopted by UniProtKB, taking into account the first Met.) Increased signaling via the EGFR pathway has been well documented in CRC and is due to upregulation of activating ligands such as EGF, epiregulin, amphiregulin, and TGF*α* or by activating mutations in EGFR itself [14]. Constitutive activation of the RAS/RAF/MEK/ERK pathway occurs in almost 50% of CRC patients due to mutations in the *KRAS* and *BRAF* genes [15–17]. It is likely therefore that phospho-K8 may be more abundant in such cases. However, little is known of the frequency and type of K8 phospho-isoforms in CRC. The aims of this study were to identify the isoforms of K8 in tumors from a 2D DIGE study, to identify the position of the modifications using mass spectrometry (MS), and to validate the overabundance of these isoforms in CRC patients' tumor relative to matched normal mucosa by western blotting. Finally, we sought to determine the effect of blocking MAP kinase activity on the level of K8 phosphorylation and level of induced apoptosis.

## 2. Materials and Methods

### 2.1. Specimens

Tumor and matched adjacent normal mucosa specimens, collected from patients undergoing colorectal tumor resection, were retrieved from a frozen tissue bank at The Queen Elizabeth Hospital. None of the patients had received prior chemotherapy or radiotherapy. Ethics approval was received from the institutional Ethics of Human Research Committee (protocol 1993/59) and informed consent was obtained in all cases. The work described has been carried out in accordance with The Code of Ethics of the World Medical Association (Declaration of Helsinki) for experiments involving humans.

### 2.2. Antibodies

Antibodies used were K8 (ab9023) at 1 : 500 dilution, K8 PS74 (ab32579), K8 PS432 (ab59434), and *β* actin (ab8227) used at 1 : 1000 (all from Abcam, Cambridge, MA, USA). K8 PS24 (EP1629Y) antibody was used at 1 : 5000 dilutions (Novus Biologicals, Littleton, CO, USA). Cleaved caspase-3 (Asp175) was used at 1 : 500 (Cell Signalling Technology, Beverly, MA, USA). Antibodies for *β* actin (sc-47778) and EGFR (sc-120) were both used at 1 : 500 (Santa Cruz, CA, USA). Cy3- (anti-mouse) and Cy5- (anti-rabbit) labelled secondary antibodies were used at 1 : 1000 (GE Healthcare, Buckinghamshire, UK).

### 2.3. Laser Microdissection (LMD) and Protein Extraction

Malignant glands and normal crypts were enriched using LMD on matched tumor-normal tissue for 2D DIGE analysis. We used our previous 2D DIGE pilot data to calculate the sample size required for statistically significant differential expression results using the method described by Hunt et al. (2005) for gel-based quantitative proteomics [18]. To show a 3-fold difference in expression, for a power of 90% and *P* < 0.05, 8 tumor and matched normal samples were needed. Frozen tissue (all TNM stage II cases) were embedded in OCT and cryosectioned (30 *μ*m) onto foil-framed PET membrane slides (Leica, Wetzlar, Germany). Sections for LMD were unfixed and unstained while adjacent sections on glass slides, fixed with 70% ethanol and stained with haematoxylin, were used for navigation purposes during LMD (Supplementary Figure  1 will be available at doi:10.5402/2012/706545). Using the Leica AS LMD6000 system (Leica Microsystems, Wetzlar, Germany), tissue was microdissected into the cap of a 0.5 mL microfuge tube containing 40 *μ*L of Thiourea/Urea/CHAPS (TUC) buffer (7 M Urea, 2 M Thiourea, 4% CHAPS, 30 mM Tris) with Halt protease and phosphatase inhibitors (Thermo Fisher Scientific, Rockford, IL, USA). After solubilisation overnight, samples were purified using a 2D Clean Up kit (Bio-Rad, Hercules, CA, USA), quantified using the EZQ protein assay (Invitrogen, Carlsbad, CA, USA) and diluted with TUC buffer to 5 *μ*g/*μ*L.

### 2.4. 2-Dimensional Difference Gel Electrophoresis (2D DIGE)

2D DIGE was performed according to manufacturer's protocol (GE Healthcare). Briefly, protein (50 *μ*g) from each of the tumor and normal samples and pooled internal reference standard was separately labelled with either Cy3, Cy2, or Cy5 CyDye DIGE fluors (GE Healthcare), combined, and 2D gel electrophoresis performed (see supplementary data for more detailed method). The pooled reference consisted of equal amounts of every sample in the cohort. Each gel was scanned (Typhoon 9400 Variable Mode Imager, GE Healthcare) to produce 3 independent spot maps corresponding to the Cy2, Cy3 and Cy labelled proteins, and the spot maps analysed with DeCyder v5 software (GE Healthcare).

### 2.5. Protein Identification

A 2D preparative gel containing 150 *μ*g of pooled unlabeled LMD tumor protein was electrophoresed as before and visualised using a MS-compatible silver stain [19]. Protein spots of interest were matched between the analytical gels and the preparative gel, excised, washed five times with 25 mM ammonium bicarbonate/50% acetonitrile, dehydrated with 100% acetonitrile, and digested with 20 *μ*L of 20 *μ*g/mL trypsin in 25 mM ammonium bicarbonate/10% acetonitrile overnight at 37°C. Digested peptides were analysed using a Dionex Ultimate 3000 HPLC (Dionex Corp, Sunnyvale, CA, USA) coupled to a Thermo LTQ XL linear ion trap mass spectrometer fitted with a nanospray source (Thermo Electron Corp, San Jose, CA, USA) as previously described [20]. Peak lists were generated with the Bioworks 3.3 software (Thermo Scientific) using the Sequest algorithm and searched against the IPI human data base v3.58. A correct match was assigned when the cross-correlation scores of matches were greater than 1.5, 2.0, and 2.5 for a charge state of 1, 2, and 3 peptide ions respectively, the peptide probability score was less than 0.001, and when 2 or more unique peptides per protein were identified.

### 2.6. Purification and Identification of K8 Phosphorylated Proteins

Protein extracted from SW480 cells (750 *μ*g) was separated by 2DE using 24 cm pH 3–7 NL IPG strips and 8–15% polyacrylamide gels as before. Gels were visualised with Coomassie blue. The 3 K8 isoforms of interest were individually excised from the gels and digested with trypsin as previously described [21]. Phosphorylated peptides were enriched using Phos-trap Titanium Dioxide (TiO_2_) magnetic beads (Perkin Elmer, San Jose, CA, USA) according to the manufacturer's protocol.

The eluted peptide solution (1.5 *μ*L) was spotted onto a 600 *μ*m AnchorChip (Bruker Daltonics, Bremen, Germany) target plate with 1 *μ*L of 2,5-DHB (10 mg/mL) matrix in 50% ACN/0.1% TFA/0.1% PA. MALDI TOF MS/MS was carried out as previously described [21] using a Bruker ultraflex III MALDI TOF/TOF mass spectrometer (Bruker Daltonics). The spectra and mass lists were exported to BioTools v3.1 (Bruker Daltonics), where the MS and corresponding MS/MS spectra were combined and submitted to the in-house Mascot database-searching engine (Matrix Science). Data was matched against the SwissProt 57.1 database. In addition, an *in silico* digest of K8 was performed using the sequence editor platform of BioTools to identify potential phosphopeptides. Precursor ions suspected to be phosphorylated peptides were chosen for MS/MS analysis. Spectra ion annotation was performed using MOWSE and probability scores. For spectra that matched to precursor ions of potential phosphorylated peptides, annotation was performed using BioTools, and each phosphorylation position was evaluated manually.

#### 2.6.1. Confirmation of K8 Phospho-Serine Isoforms

2D western blots of protein extracted from the CRC cell line Caco2 were performed with phospho-specific antibodies against PS24, PS432, and PS74. Protein (300 *μ*g) was separated by 2DE as before and transferred using a semidry apparatus for 1 hour at 70 mA per blot onto low fluorescent PVDF membrane. Membranes were blocked in 5% skim milk PBST, washed, and incubated overnight at 4°C with each of the primary antibodies. Membranes were washed and incubated in the dark at room temperature for 1.5 hours with ECL Plex fluorescent detection Cy3 antibody (GE Healthcare) at a 1 : 1000 dilution. Membranes were washed, air dried, and imaged using a Typhoon 9400 Imager (GE Healthcare).

#### 2.6.2. Quantification of Phospho-K8 Isoforms by Western Blotting

To determine the levels of K8 and the phosphorylated isoforms, western blotting was performed on 30 tumor and 30 matched normal mucosal samples (10 stage I, 10 stage II, and 10 stage III cases). For protein extraction, 30 *μ* cryosections were pulverised in a mortar and pestle under liquid nitrogen and placed in 200 *μ*L of sample buffer (2% SDS, 10% glycerol, 62.5 mM TrisHCl pH 6.8, 6 M urea, 1x Halt phosphatase inhibitors (Sigma), 65 mM DTT, and 150 U Benzonase (Sigma)). Samples were purified using the 2D Clean Up Kit (GE Healthcare), resuspended in 100 *μ*L of sample buffer, and quantified using the EZQ assay (Invitrogen). Western blotting for the 3 phospho-antibodies was performed as before except that 50 *μ*g of each sample was run on 4–20% polyacrylamide gels. Unmodified K8 was also detected using a K8 antibody. A *β* actin loading control antibody was included for all blots. Membranes were incubated with ECL Plex Cy3 and Cy5 antibodies (GE Healthcare) and imaged as before. Bands were quantified using ImageQuant V5 software.

Levels of K8 were normalised to *β* actin in each sample and the ratio of tumor to normal protein expression was calculated. For the K8 phospho-specific antibodies, each band was normalised to *β* actin, then normalised to total K8 expression. The ratios of the level of phospho-isoforms in tumor versus normal were calculated. Statistical analysis was performed using GraphPad Prism 4 software.

### 2.7. Blocking EGFR Signaling in Caco2 Cells

The colon cell line Caco2 (ATCC, Manassas, VA, USA) was treated with the sc-120 antibody (Santa Cruz) to block the ligand binding site of EGFR. Caco2 cells were plated in duplicate at 1 × 10^5^ cells per well in a 24-well plate in a total volume of 350 *μ*L RPMI media with 10% FCS in humidified 5% CO_2_ in air. Cells were treated with 1 *μ*g of antibody per well. This amount was determined to be saturating by flow cytometry (Supplementary Figure  2). Treated and untreated control cells were harvested at 4, 6, and 8 hours after antibody treatment by lysis in 50 *μ*L of sample buffer. Lysates were quantified and western blotting was performed on 25 *μ*g of each sample for K8, PS24, PS74, PS432, and a *β* actin loading control as before. For the K8 phospho-specific antibodies, each band was normalised to *β* actin, then normalised to total K8 expression. This experiment was repeated three times and the statistical significance of the difference between means for each treatment time was determined by ANOVA, with *P* < 0.05 being considered significant.

### 2.8. Apoptosis Induction of sc-120 Antibody-Treated Cells Compared to Untreated Cells

Caco2 cells were treated with the sc-120 antibody as before to reduce K8 PS74 and PS432 levels. Six hours after sc-120 treatment 1 *μ*M of doxorubicin (DOX) was added to the appropriate treatment wells as a cell stressor. Treatments were sc120 plus DOX, sc120 alone, DOX alone, and untreated cells. After 8 hours, cells were washed with PBS and lysed in 50 *μ*L of sample buffer. Lysates were quantified and western blotting was performed on 50 *μ*g of each sample. Fluorescence intensity of bands was quantified as before. Levels of cleaved caspase-3 were normalised to *β* actin expression in each sample. This experiment was repeated three times and the statistical significance of the difference between means for each treatment time was determined by ANOVA, with *P* < 0.05 being considered significant.

## 3. Results

### 3.1. Laser Microdissection and Protein Extraction

To avoid contamination from other cell types, LMD was used to enrich for epithelial cells from matched tumor-normal paired tissues from 8 stage II cases. The mean tumor protein yield was 115 ± 29.4 *μ*g, from an area of 47 ± 7.9 mm^2^ (2.4 *μ*g/mm^2^) while the mean normal mucosa protein yield was 91 ± 10.1 *μ*g, from an area of 60 ± 28.9 mm^2^ (1.5 *μ*g/mm^2^).

### 3.2. Determination of Differentially Expressed Proteins by 2D DIGE and Mass Spectrometry

**Figure 1:**
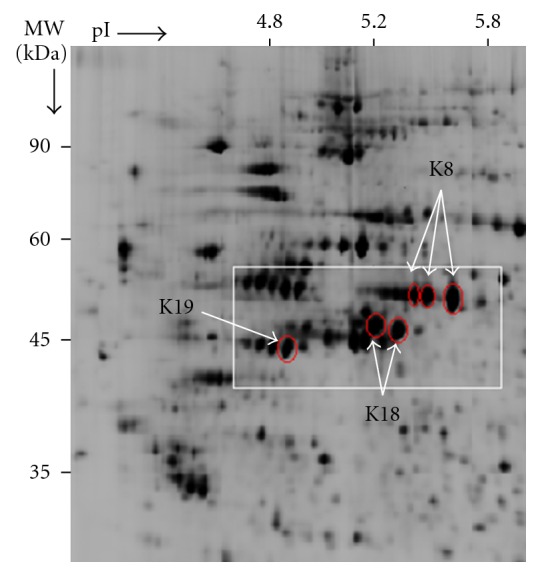
A DeCyder 2D gel image displaying the keratin proteins significantly increased in the tumors. Circled are the identified keratin proteins significantly increased in the LMD tumors compared to matched normal mucosa. The labeled protein spots in the boxed area correlate with the data given in Tables 1 and 2.

Using DeCyder v5 software, a total of 1,745 protein spots were matched across all gels and the tumor-normal abundance ratio of each protein spot listed with its significance (Student's *t*-test). The data set was filtered by selecting protein spots with an average abundance ratio of tumor to matched normal of ≥2-fold, limited to spots matched on all gels and applying a significance threshold of *P* ≤ 0.05. The protein with the highest level of tumor upregulation was K8 and was among 25 proteins significantly upregulated by ≥2 fold between tumor and matched normal mucosa, identified by ion trap MS (data not shown). Three protein spots were identified as K8, 2 spots as K18, and a further spot corresponding to K19 (Figure 1 and Table 1). All 3 K8 protein spots were increased ≥2-fold in 6/8 tumors. The average tumor to normal abundance ratios for the isoforms of K8 calculated by DeCyder software are shown in Table 2.

### 3.3. Phosphorylation Site Identification

The K8 isoforms detected as increased in tumor were excised from a preparative 2D gel and digested with trypsin. Phosphorylated peptides were enriched using Phos-Trap TiO_2_ beads and subjected to MALDI-TOF/TOF MS analysis. The annotated MS spectra pre- and postbead enrichment is shown in Supplementary Figure  3. Precursor ions with *m*/*z* ratios of potential phosphopeptides were chosen for MS/MS analysis. Two precursor ions at *m*/*z* 991.378 and 3805.269 were identified in the K8 isoforms, and matched to previously annotated phosphopeptides by performing an *in silico* digest of K8 using the sequence editor platform of BioTools (Bruker Daltonics). Complete y-ion sequence coverage was obtained for the [M+H]^+^-ion 991.378 ([23]–[37] SYTSGPGSR), with the loss of 80 and 98 being detected (Supplementary Figure  4). Partial sequence and the mass loss of a phosphate were obtained from the [M+H]^+^-ion 3805.269 ([415]–[454] TTSGYAGGLS(s)A(y)GGL(t)(s)PGLSYSLGSSFGSGAGSSSFSR) (Supplementary Figure  5). The residue that had lost the phosphate group was assigned by using BioTools, where all possible phosphorylation sites within the peptide were analysed and ranked according to probability. The peptide contains 4 potential phosphorylation sites according to the Uniprot data base (http://www.uniprot.org/uniprot/P05787) of which 2 have been assigned by similarity and 2 by annotation. The serine residue 432 was scored as having the highest probability of phosphorylation using BioTools. Using phospho-specific antibodies, 2D western blots confirmed the presence of the serine phosphorylated residues 24, 432, and 74 in the K8 spots of interest (Supplementary Figure  6).

### 3.4. Quantification of Phospho-K8 Isoforms by Western Blotting

**Figure 2:**
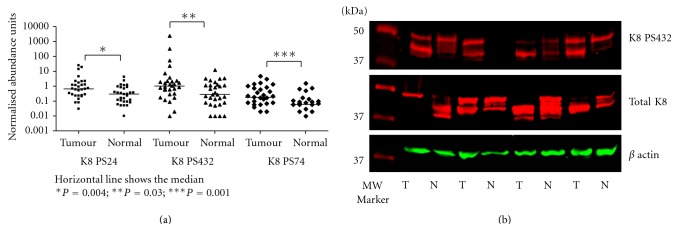
Levels of K8 phospho-isoforms determined by western blotting. (a) Plots showing abundance levels of PS24, PS432, and PS74 normalised to *β* actin loading control and total K8 in 30 matched pairs of tumor-normal tissues. (b) A representative western blot of total K8, *β* actin and PS432. In the case of multiple bands, all bands were included in quantification. Tumor (T) and normal (N) pairs were loaded in adjacent lanes from left to right. Statistical analysis was performed using a 2-tailed Wilcoxon matched pairs test.

**Figure 3:**
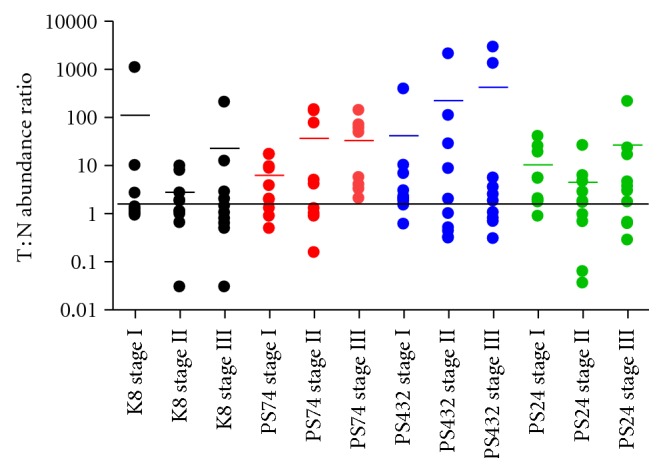
Tumor : normal abundance ratios according to stage. Graph shows the abundance ratio tumor (T) to matched normal mucosa (N) for total K8 and each phospho-serine K8 isoform, according to tumor stage. Horizontal line shows the 2-fold abundance ratio T : N. There was no significant difference in the means for each isoform abundance ratio among different stages.

The results of western blot quantification are shown in Figure 2(a) with a representative blot shown in Figure 2(b). Multiple bands for K8 were detected in some samples within the molecular weight range of 40 to 55 kDa (Figure 2(b)) indicating proteolytic cleaved forms of K8. Such proteolysis of K8 has been previously described in CRC [22]. The range of MW for K8 detected on western blotting was not found on the 2D DIGE gel (Figure 1). The reason for this could be due to either the cleaved forms resulting in a shift in isoelectric point and so were not present in the identified spots, or that they were present in too low abundance relative to the intact isoforms to be detected by DIGE. The median expression level (arbitrary units) of total K8 in the tumor samples was 2.75 compared to 1.8 in the matched normal samples (*P* = 0.053 Wilcoxon signed rank test). For the phospho-K8 isoforms the median level between the tumor and matched normal was significantly different: PS24 0.667 and 0.29, respectively, *P* = 0.004; PS432 1.02 and 0.30, respectively, *P* = 0.03; PS74 0.18 and 0.06, respectively, *P* = 0.0005 (Wilcoxon signed rank test). Among the 30 patients, 16/30 (53%) showed overabundance (tumor : normal ratio ≥2) for PS24, 17/30 (57%) for PS432, and 19/30 (63%) for PS74 (Figure 3).

### 3.5. Blocking EGFR Signaling in Caco2 Cells Decreases Levels of PS74 and PS432

**Figure 4:**
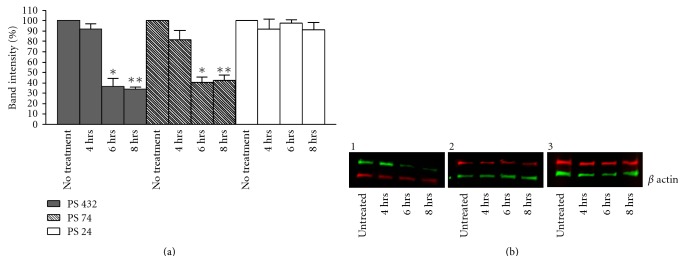
Blocking EGFR signaling in Caco2 cells leads to reduction in PS74 and PS432 levels. Caco2 cells were treated with the EGFR ligand binding blocking antibody sc-120 for 4 hours, 6 hours, and 8 hours (*n* = 6 per treatment group). (a) Western blotting and band intensity quantification was performed on the cell lysates with antibodies against total K8, PS24, PS74, and PS432. Levels of PS74 and PS432 were significantly reduced 6 and 8 hours after sc-120 treatment, *P* < 0.0001. As expected, levels of PS24 remained unchanged by treatment with sc-120, *P* = 0.73. (b) Representative western blots: B1, PS432; B2, PS74; B3, PS24. The lower bands in each case are the *β* actin loading controls.

Caco2 cells, wild type for BRAF and KRAS, were treated with the sc-120 antibody (Santa Cruz) that blocks ligand binding and hence activation of EGFR. Treated and untreated cell lysates were then analysed by western blotting for PS24, PS74, and PS432. Treatment of Caco2 cells with the sc-120 antibody led to a significant decrease in the levels of PS74 (*P* < 0.0001) and PS432 (*P* < 0.0001) (ANOVA) at 6 and 8 hours after treatment (Figure 4). At the 4-hour time point levels of PS74 were 81.5%  ± 8.8 that of the untreated cells. At the 6 and 8 hour time points PS74 levels had decreased to 40.7%  ± 5.1 and 42.3%  ± 5.3 that of the untreated cells, respectively. Levels of PS432 were 91.7%  ± 5.2 that of the untreated cells at 4 hours, and decreased to 36.7%  ± 7.5 and 34.0%  ± 2.1 of the untreated cells at the 6 and 8 hour time points, respectively. PS24 levels were not significantly different after treatment with the sc-120 antibody, *P* = 0.73.

### 3.6. Caco2 Cells with Decreased Levels of PS74 and PS432 Showed Increased Apoptosis Following DOX Treatment

**Figure 5:**
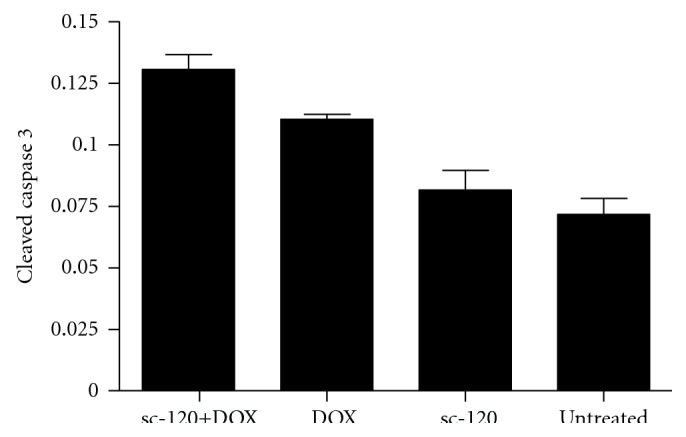
Apoptosis assay (cleaved caspase-3). Cleaved caspase-3 levels were significantly higher in the sc-120 + DOX-treated cells compared to the DOX-alone, sc-120-treated, and -untreated cells, *P* = 0.0005 (ANOVA).

K8 PS74 and PS432 levels were reduced by inhibition of EGFR signalling using the sc-120 anti-EGFR antibody, following which they were treated with DOX to induce apoptosis. Apoptosis induction was analysed by western blotting for cleaved caspase-3. Cleaved caspase-3 levels were significantly higher in the sc-120 antibody + DOX-treated cells (0.13 ± 0.006) compared to the DOX alone (0.11 ± 0.002), sc-120-treated (0.081 ± 0.008) or -untreated (0.071 ± 0.006), *P* = 0.0005 (ANOVA) (Figure 5). The results represent an increase in cleaved caspase 3 levels relative to untreated cells of 82%, 54%, and 14%, respectively.

## 4. Discussion

This study found K8 isoforms, CK18, and CK19 to be significantly upregulated in laser microdissected tissue from 8 stage II tumor samples compared to matched normal by 2D DIGE. The advantage of 2D DIGE over standard 2D gels is the greatly improved statistical validity obtained due to the presence of the tumor, normal and pooled reference sample, to which abundance of each spot is normalised, on every gel. Spot matching within a gel is therefore exact. The software uses a novel algorithm used to match spots gel to gel and, through spot normalisation, greatly reducing gel-gel variation effects and increasing the statistical value of results. We hypothesised that the K8 isoforms present in abundance in the tumor samples compared to matched normal were a result of increased phosphorylation. Indeed, this was confirmed using mass spectrometry, bioinformatics, and 2D western blotting. In a larger cohort of human colon cancer tissues, levels of all 3 K8 isoforms PS24, PS432, and PS74 were found to be significantly increased in tumor compared to matched normal mucosa across all stages.

Mutation of genes in the EGFR signaling cascade results in increased RAS/RAF/MEK/ERK pathway activation and increased MAPK (ERK1/ERK2) activity in particular [23]. The kinase ERK1 is known to phosphorylate K8 serine 431 [24]. It has also been reported that ERK1 is involved in phosphorylation of K8 at serine 73 in A549 tumor cells in response to an apoptosis-inducing agent, as the phosphorylation was blocked only by an ERK inhibitor [25]. We showed that blocking of the EGFR-ligand binding site in the CRC cell line Caco2 (wild type for both KRAS and BRAF) resulted in a significant decrease in not only K8 PS432 but also PS74 (*P* < 0.0001) in support of the earlier work. The MAP kinase p38 and JNK are also known to phosphorylate serines 431 and 73 [10, 13], but the blocking of EGFR would have had no direct effect on the activity of these kinases.

In our study the level of K8 PS24 was significantly increased in the tumor samples compared to matched normal (*P* = 0.004). Treatment of Caco2 cells with the sc-120 antibody did not significantly affect K8 PS24 levels, suggesting that kinases involved in the EGFR signaling pathway are not involved in the phosphorylation of this residue. One study has shown that protein kinase C *ε* phosphorylates K8 S24 in rat pituitary cells stimulated by thyrotropin-releasing hormone [26].

This study in colon cancer has shown that the majority of cases show increased tumor levels of PS74, PS24, and PS432. Decreasing the levels of PS74 and PS432 levels in Caco2 cells resulted in the cells being more susceptible to apoptosis induction following DOX treatment. Phosphorylation of K8 residues causes disaggregation of K8 IF, increased solubility and intracellular redistribution [9, 27–29]. The soluble form of K8 acts as a phosphate “sponge” inhibiting phospho-kinase activation of proapoptotic substrates and thereby protecting the cells from apoptosis [11]. K8 hyperphosphorylation at PS74 and PS432 would thus promote tumor cell survival and progression and may be a biomarker for colon tumors with increased resistance to apoptosis-inducing therapeutic agents. However, longitudinal studies in a larger patient cohort are needed to address this point.

## Supplementary Material

We have provided 6 supplementary figures illustrating (1) laser microdissection of tumour section, (2) flow cytometry of Caco2 cells to determine saturating amount of anti-EGFR SC-120 antibody, (3) MALDI-TOF MS spectra showing detection of PS432 after Phos-Trap™ enrichment, (4) Tandem MS analysis showing a mass loss of 98 (a phosphate group) conferring to K8 PS74, (5) Tandem MS analysis identifying K8 PS432 as the most likely residue, (6) 2D western blots confirming the phosphorylated serine residues of K8 isoforms as PS24, PS432 and PS74. Finally, the 2D DIGE method is described in further detail.

## Figures and Tables

**Table 1:** Protein identification.

Spot	ID^a^	Acc no.^b^	pI^c^	MW (Da)^d^	Xcorr^e^	No. of peptides^f^	% Sequence coverage^g^
1	K8	P05787	5.38	53672	270.2	19	39.75
2	K8	P05787	5.50	53672	230.3	16	39.13
3	K8	P05787	5.70	53672	210.3	15	34.78
4	K18	P05783	5.21	48029	100.3	9	30.39
5	K18	P05783	5.30	48029	120.2	8	31.40
6	K19	P08727	4.90	44065	180.3	14	53.50

^a^Protein identified.

^b^SwissProt accession number.

^c^Calculated isoelectric point.

^d^Calculated molecular weight, Daltons.

^e^Xcorr: significance score.

^f^Number of peptides sequenced.

^g^% of the full length protein sequence covered by identified peptides.

**Table 2:** Quantitative 2D DIGE data (DeCyder software).

						Individual tumour-normal abundance ratios					
Spot	ID	Ave. ratio^a^	*P* ^ b^	1	2	3	4	5	6	7	8

1	K8	4.56	0.00026	2.50	8.70	3.50	6.20	5.20	5.10	1.30	3.90
2	K8	4.36	0.0052	1.30	7.50	4.30	7.50	3.40	7.70	0.80	2.00
3	K8	3.98	0.0019	1.40	4.50	2.70	4.40	5.30	5.70	0.95	6.80
4	K18	4.22	0.013	1.20	10.50	4.40	2.00	4.60	8.50	1.60	0.80
5	K18	4.00	0.037	0.70	9.60	4.20	3.00	4.60	7.70	1.70	0.48
6	K19	2.34	0.018	0.93	4.32	1.71	2.01	1.83	4.96	0.87	2.13

^a^Average tumor-normal abundance ratio.

^b^Significance value, Student's paired *t*-test.
