# Supplementary material for: Increased Phospho-Keratin 8 Isoforms in Colorectal Tumors Associated with EGFR Pathway Activation and Reduced Apoptosis
Source: ISRN Mol Biol. 2012 Jan 31;2012:706545. doi: 10.5402/2012/706545 (PMC4908239; doi:10.5402/2012/706545)
Supplement: Supplementary file 1 — We have provided 6 supplementary figures illustrating (1) laser microdissection of tumour section, (2) flow cytometry of Caco2 cells to determine saturating amount of anti-EGFR SC-120 antibody, (3) MALDI-TOF MS spectra showing detection of PS432 after Phos-Trap™ enrichment, (4) Tandem MS analysis showing a mass loss of 98 (a phosphate group) conferring to K8 PS74, (5) Tandem MS analysis identifying K8 PS432 as the most likely residue, (6) 2D western blots confirming the phosphorylated serine residues of K8 isoforms as PS24, PS432 and PS74. Finally, the 2D DIGE method is described in further detail. [file 706545.f1.doc]

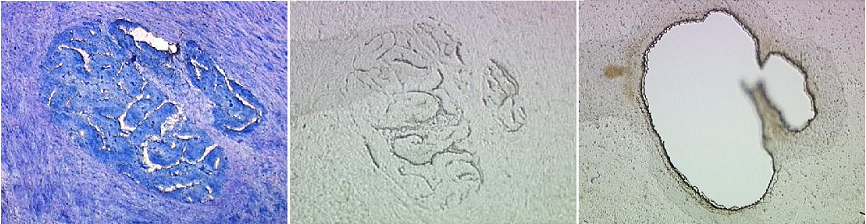


**a**

**b**

**c**

**Supplementary Figure 1**

Laser microdissection (LMD): Representative images of fixed and stained tumour tissue section (a), unfixed and unstained adjacent section prior to LMD (b), and after LMD (c). Arrow points to a malignant crypt.


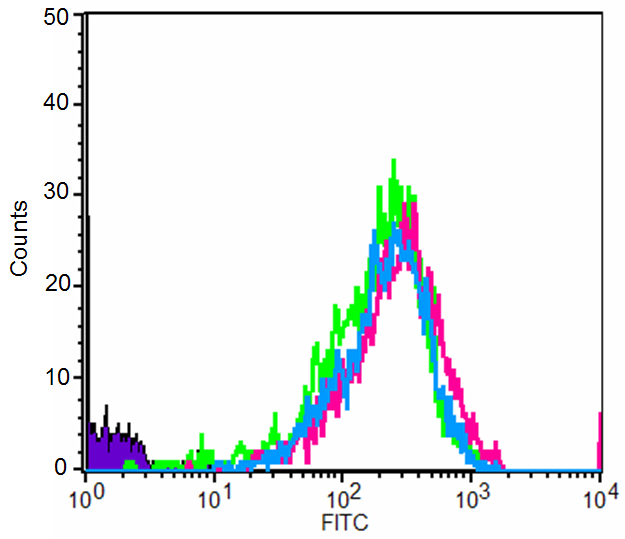


Key

Name

Parameter

Gat

Isotype control ab

FL1-H

G1

0.5 ug ab SC-120

FL1-H

G1

1.0 ug ab SC-120

FL1-H

G1

2.0 ug ab SC-120

FL1-H

G1

**Supplementary Figure 2**

Flow cytometry to determine the amount of sc-120 antibody to saturate the surface of the Caco2 cells. One microgram of antibody per 1 x 10^5^ cells was selected as saturating.

1419.838

1129.671

1030.610

1797.964

906.491

1277.777

2004.069

2455.504

2109.188

1224.666

979.502

2288.294

3806.123

2547.472

3340.039

1507.800

2050.208

732.230

1637.000

3168.798

Digest prior to enrichment

0

0.5

1.0

1.5

1797.814

991.378

2124.959

1308.641

3805.269

1079.500

733.885

899.399

3339.408

1925.900

2455.190

2211.032

3168.268

Phos-Trap™ enriched

digest

3

2

1

1000

1500

2000

2500

3000

3500

m/z

PS432: [415] – [454]

PS24: [23] – [31]

Intensity x10^4^ [a.u.]

**Supplementary Figure 3**

MALDI-TOF MS spectrum: stock K8 trypsin digest prior to phosphopeptide enrichment (top), and after phosphopeptide enrichment (bottom) using Phos-Trap™ beads. The precursor ions of the peptides containing the phosphorylated residues PS24 and PS432 can be seen in the enriched MALDI-TOF spectra at m/z 991.378 and 3805.269 respectively.

**Supplementary Figure 4**

Tandem MS analysis of [M+H]+: 991.4 Da. (SYTSGPGSR)

A mass loss of 98 from the terminal Y ion series serine residue was detected, indicating the loss of a phosphate group. This phosphorylated peptide, conferring to phosphoserine 24 based on the y8 ion at m/z 824.5 and y9 ion at 991.4, was detected in the most acidic K8 isoform by MALDI TOF/TOF MS.

**Supplementary Figure 5**

Tandem MS analysis of [M+H]+: 3805.3 Da. (TTSGYAGGLS(s)A(y)GGL(t)(s)PGLSYSLGSSFGSGAGSSSFSR)

Mass losses of 80 and 98 from the Y ion series were detected indicating the loss of a phosphate group. Residues potentially phosphorylated are highlighted in brackets. Using BioTools and manual annotation, the most probable phosphorylated residue was calculated to be PS432.


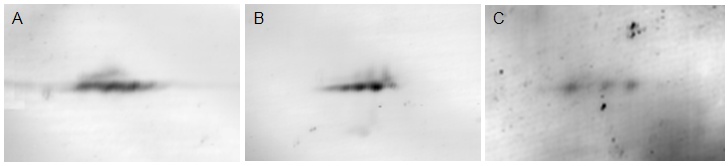


**Supplementary Figure 6**

2D western blots confirming the phosphorylated serine residues (A) 24, (B) 432, and (C) 74 are present in the 3 K8 isoforms detected in higher abundance in tumors relative to matched normal mucosa.

**2D DIGE Method**

An internal standard was made by pooling 25 µg of each of the tumor and normal protein samples and 400 µg labelled with 3200 pmol of Cy2 dye (GE Healthcare) as per the recommendation. Protein (50 μg) from the 8 tumor and 8 normal samples was separately labelled with either Cy3 or Cy5 and dye-swaps were performed. Labelled matched pairs of tumor-normal protein were mixed with 50 µg of the labelled internal standard, and made up to 450 µl in TUC buffer with DTT to 65 mM and 3-7 Ampholytes to 1%. Samples were re-hydrated overnight into in 24 cm pH 3-7 non-linear IPG strips (GE Healthcare) at 50 V, 20°C, followed by isoelectric focusing for approximately 80,000 Vhrs, 20°C, using IPGphors (GE Healthcare). Second dimension 8-15% gradient SDS PAGE was performed at 350 V using an Ettan DALTsix Vertical electrophoresis system (GE Healthcare). Imaging of Cy2-, Cy3- and Cy5-labelled protein spot maps was performed (Typhoon 9400 Variable Mode Imager, GE Healthcare) and the gel images cropped (ImageQuant v5.2 software, GE Healthcare) and analysed with DeCyder™ v5 (GE Healthcare).
